# Diagnosis and Treatment of Root Canals*Abstract from a paper read before the New York Second District Dental Society and printed in the July *Dental Items of Interest*.

**Published:** 1918-08

**Authors:** Edgar D. Coolidge


### DIAGNOSIS AND TREATMENT OF ROOT CANALS*

BY EDGAR D. COOLIDGE, D.D.S.

Much importance should be placed upon the diagnosis in every case. Where a vital pulp is to be removed, a radiograph should be taken to reveal the condition, number, size and direction of the root or roots. I believe that where a root is very crooked or constricted, or gives evidence by radiograph of uncertainty in the result the pulp should be retained if there is any possibility of retaining it.

In the interpretation of radiographs of teeth having periapical involvement there are many difficulties to contend with, such as determining the exact extent of an area of absorbed bone, which interpretation is based on the shadow cast upon the film. There is more or less difficulty in some cases in correctly determining whether such an area is partly caused by the normal bone canals and foramina or whether it is entirely pathologic; and again, *it is*

---

* Abstract from a paper read before the New York Second District Dental Society and printed in the July *Dental Items of Interest.*

*possible to overlook* such an area where a very heavy plate of bone exists on both labial and lingual sides, even though the softer cancelous bone between these two plates may be absorbed away to some extent. There is very little doubt that some of these conditions are overlooked. Again there may be definite evidence in the radiograph of an area or cavity, and the tooth over which such is found may contain a vital pulp. It is extremely difficult in some cases to follow the course of a sinus through the bone from its source, and the radiograph may appear deceiving and may confuse one's diagnosis. Clinical evidence is quite essential to correctly locate these conditions and to find the diseased tooth. It is of great value in the diagnosis if it can be determined whether a cavity in the region surrounding a root envelopes or lies back of it or in front of it, and whether there is much of the peridental tissue lost, denuding the cementum. This is of considerable importance in the prognosis of the case, and I believe the explanation of why a cure can be effected in one case and not in another which looks very much the same in the radiograph, is because in one the cementum is actually denuded and infected, and only apepars to be so in the other.

A similar situation is seen in the treatment of certain cases of suppurative pericementitis, where a pocket, or seemingly detached tissue, appears to become reattached after treatment contrary to the known principles in regard to reattachment to denuded and pus infected cementum.

Another valuable point in the diagnosis is the determining from the radiograph whether an infection has been walled off by the natural defenses of the body, or whether the condition is still developing. It is also valuable in the consideration of a case to observe whether the area has a definite line of demarcation from the healthy tissue or whether it gradually fades away until it is lost in the shadow of the healthy bone. It is impossible to determine the relative danger of systemic infection from these various conditions, but there is little doubt of the presence of streptococci in almost every one, and every one contains other forms of micro-organisms.

The limitations of radiographic diagnosis should always be considered and a careful survey of the clinical evidence should be an important factor in determining the condition and prognosis of each case.

In discussing the treatment of these cases, I do not wish to convey the impression that there is only one method by which successful results can be obtained, but I desire to give the result of my own observation and experience.

In the treatment of clean cases the best opportunity for success is offered and the case should be handled with the greatest care and skill to thoroughly remove the organic matter without destruction of the periapical tissue. In the process of pulp removal the safest method to follow in desensitizing is local anesthesia by novocain, because I believe the usefulness of novocain or cocain in pressure anesthesia has not become ancient history; neither do I think that the devitalization method by the use of arsenic should be relegated to the past. When arsenic is to be used, it should always be kept in mind that the vitality of the pericemental tissue is endangered by too long an application, and it should only be used as an obtunding or desensitizing agent to enable subsequent exposure of the pulp and its removal by novocain or cocain.

After desensitizing and a complete exposure of the pulp, by which I mean the gaining of sufficient access to see the mouth of each canal and to eliminate every curve encountered that is possible to eliminate by cutting away the crown with a bur, one is confronted with the entering and cleaning of the canals to the foramina without making obstructions which prevent a successful operation. The one principle that has given the greatest success in my operations in canals is to carefully *insert the broach to the apical foramen first and continue* the enlarging and cleaning *from*

*that point out* by a process of filing, curettment and raking with the graded sizes of broaches, always carrying on an up and down movement and never a turning or twisting more than a half revolution in one direction, and back the other way the same distance. There are several difficulties usually encountered, among which are the packing of debris and pulp tissue ahead of the broach, but this can be avoided if care is used. Another difficulty is in losing the canal after working for a time. This can usually be avoided if the broach is selected each time which will readily pass to the apical foramen.

The use of strong alkalies and acids are very dangerous, although there is little chance of mechanically removing all the organic matter from all the branches of canals similar to those shown in the illustrations of teeth by Dr. Callahan. At the same time I think the danger of injury to the periapical tissue is so great when a persistent effort is made to remove this by chemical means that it overbalances the benefit derived in a large number of cases. There must be some means found of eradicating this inaccessible tissue or compensating for it with means that are nondestructive to the vital tissue at the apex, before we shall have completely solved the problem of root canal treatment. That there is a large percentage of teeth having the multi-canaliculated and multiple foraminated roots I am convinced, but I am not willing to admit that these can all be cleaned and filled thoroughly and completely by any present method of operation published today, without injury to the periapical tissue. I do maintain that if this class of cases is treated and filled by means which do not injure the periapical tissue and if they show radiographic evidence of being filled completely to the main foramina without excess, that we have done the best that can be done at the present time, and the prospect of future infection and pathologic disturbance about the apices of teeth so treated, is very remote.

In treating infected canals where there is no visible destruction of tissue about the periapical region, we are confronted with the difficulty of disinfecting the root without involving the soft tissue either by means of the infection or the disinfectant used. This group contains the teeth with dead pulps and those where previous imperfect operations have been performed. One of the most unfortunate things encountered with these teeth is the partial canal filling of cement. It is extremely difficult and hazardous to remove a cement filling from a root canal. Frequently pieces of metal are encountered also, which are very troublesome and sometimes impossible to remove.

The antiseptics which may be used in treating these roots without danger to the periapical tissue are limited. The greatest factor in cleaning these canals is the mechanical filing and curetting done in the process of enlargement. All strong chemical agents introduce danger to the surrounding tissue. The destructive action of formaldehyd has become recognized and it is generally accepted that it should never be allowed to come in contact with healthy tissue. This principle precludes the use of this agent in more than the first or second treatment of these cases, when a sufficient amount of decomposed tissue remains near the foramen to prevent the escape of this penetrating and irritating gas. The acid and alkali treatment is safe to within a short distance of the foramen, but here again great danger enters when these destructive agents are used. It seems that the only safe method lies with nondestructive agents supplemented by a thorough curettment.

The possibilities of ionization of a sodium chloride solution in the root canal is worthy of consideration, though it has not gained a general popularity. By means of this treatment it is claimed that the dentin can be disinfected for a depth of one milimeter, without danger to the surrounding tissue, and if this is used just previous to the filling of the canal, it is doubtless a valuable asset in freeing the canal from infection. The experience I have thus far gained in carrying on bacteriologic tests in infected cases by means of medication and by ionization, does not show any great advantage in the use of ionization, but the results of further investigation now under way may be different.

The treatment of canals with periapical infection and bone absorption is very similar to that just described. There are as great responsibilities upon us here in the determination of the advisability of extraction before a long, uncomfortable and expensive treatment is given, as in the matter of the treatment involved. It is possible to see regenerated tissue fill in these rarified areas after treatment and filling, but a picture of such a condition does not prove the absence of infection. There are certain conditions where extraction is positively indicated and these can largely be determined by the extent of denuded cementum of the root shown in the radiograph. Where a denuded root end projects into an open cavity there is little chance for a cure by medication. Root resection or extraction is indicated.

Any case showing positive destruction of the peridental membrane, denuding an appreciable area of cementum should be looked upon as serious, and so should an area showing a clear-cut outline between the healthy and the diseased tissue. Areas with no definite outline may have liquified tissue extending in radiating directions from the apex of the root and many spicules of diseased bone remain which can not be removed in any way but by extraction or curettment. Pus and micro-organisms are always found in these cavities and I have not always been able to free them from this condition by medication or ionization, and I believe many that have been considered as cured in the past, would give positive evidence of infection and pus at the apex if opened today, even though the radiograph may seem to show a regenerated tissue.

Every tooth which can be freed from pus and infectious material and which can be retained with safety to the health of the patient should be saved, but where there is much doubt as to the permanence of such a cure on account of denuded cementum, it is better to advise extraction instead of treatment. There are other conditions which prevent a successful operation, such as obstructions which can not be overcome in root canal treatment and filling, which the operator should be keen enough to recognize at the beginning; and the patient advised as to the doubtful outcome of the operation, for justice is due to both patient and operator.

Much has been left out of this discussion in regard to details of treatment which are of the greatest importance to a successful operation. The question of asepsis and sterilization has been entirely omitted, but I wish to record one statement which perhaps will convey the idea without detailed description. Surgical cleanliness and asepsis is the motto, with no dressing, instrument or material unfit for any surgical work. I wish here to pay tribute to the valuable contribution to the methods of asepsis and sterilization which have been suggested by Buckley, Best, Rheim, Ottolengui and Callahan.

The question of a successful root canal operation is largely solved when the canal is ready to be filled, and yet there remains ample opportunity for failure or unfortunate results. A canal filling which is incomplete can be removed and another attempt made, while an excess of material can only be removed by surgical means or left in the tissue. Only the canal filled to the apex and not beyond can be looked upon as perfect, although in certain cases it may be desirable to have a slight protrusion of the fluid portion of the filling. It is my desire in this work to allow only a sufficient amount of the fluid, which is chloro-percha, to pass beyond the foramen to give evidence by radiograph of completeness. In canals having large foramina it is impossible to regulate the excess unless a definite amount of material is used, and the less fluid material is used the less uncertainty will be encountered.

When the canal is ready for filling, the No. 34 S. S. White or No. 1 Kerr root canal plugger will pass to within two millimeters of the foramen of the canal, which has previously been thoroughly cleaned and measured by the measurement wire radiographed in position in the root. After thoroughly dehydrating the canal it is moistened with eucalyptol and followed by a single drop of chloro-percha carried to the apex. A piece of gutta-percha cone is cut one millimeter more than long enough to equal the length of the canal when placed upon the end of the No. 34 canal plugger, and of a size in diameter great enough not to pass through the foramen. When this is inserted and packed into the canal previously moistened with eucalyptol and a drop of thin chloro-percha, the space beyond the plugger about two millimeters in length, into which is packed the piece of gutta-percha three millimeters long, with a liquid chloro-percha to be forced in front and laterally, the chance of having too great an excess is reduced to the minimum, while there is frequently found by radiograph evidence that two or more branches of the main canal are filled at the same time. The canal is filled by packing small pieces in the same manner, one by one, until finished.

Small canals should be enlarged to insure proper filling and their filling then becomes a repetition of the same process as used in large canals. Where the apical foramen can not be passed by a broach, the method of dissolving cone after cone with the pumping motion in the canal, in a thin solution kept thin while the pumping is continued and afterward packed down by pluggers, carrying short pieces of gutta-percha cones, is recommended.

In conclusion may I call attention once more to the all-important, but, I am sorry to add, often neglected feature of root canal operations, which is surgical cleanliness, of a practical nature, which can be used by every operator. The use of destructive agents should be attended with the greatest care to preserve the health of the periapical tissue. A careful diagnosis and prognosis should be made of every case and the patient instructed accordingly, to do justice to the patient and to protect the operator from criticism when all that human hands can do with present methods of operating does not give a perfect result. Accurate measurements should be made of length, size and diameter of foramen of every canal before filling, and the operator should limit the uncertainty in filling as much as possible by the use of these measurements and the control of his filling material.

